# Cells and Cytokines in Milk of Subclinically Infected Bovine Mammary Glands after the Use of Immunomodulatory Composition GLP 810

**DOI:** 10.1155/2020/8238029

**Published:** 2020-03-22

**Authors:** Gundega Gulbe, Māra Pilmane, Vaira Saulīte, Simona Doniņa, Jevgenijs Jermolajevs, Lilija Peškova, Anda Valdovska

**Affiliations:** ^1^Riga Stradins University, Institute of Microbiology and Virology, 5 Ratsupites Street, Riga LV-1067, Latvia; ^2^Riga Stradins University, Faculty of Medicine, Institute of Anatomy and Anthropology, Anatomical Theatre, 9 Kronvalda Bulvaris, Riga LV-1010, Latvia; ^3^Latvia University of Life Sciences and Technologies, Faculty of Veterinary Medicine, Institute of Food and Environmental Hygiene, 8 Kr. Helmana Street, Jelgava LV-3004, Latvia; ^4^Latvia University of Life Sciences and Technologies, Scientific Laboratory of Biotechnology, 1 Strazdu Street, Jelgava LV-3004, Latvia

## Abstract

The aim of this study was to investigate the effect of intramammary infusions of natural composition GLP 810 with immunomodulating properties on the local nonspecific cellular and humoral immune response in cows with subclinical mastitis. The composition GLP 810 consists of lactic acid, lysozyme, glycopeptides, and 0.9% solution of NaCl. The following parameters were studied: (1) leukocyte differential distribution in milk, (2) expression of cytokines in milk leukocytes, (3) antibacterial activity, and (4) milk quality. Nineteen mammary glands in five lactating cows were infused with 10 mL of GLP 810, and nineteen other glands from five control cows were treated with 10 mL 0.9% NaCl. The results showed that after intramammary administration of the composition GLP 810 three times with 48 h intervals, the following effects on leukocyte populations in milk were observed: (1) an increase in the number of polymorphonuclear leukocytes and lymphocytes and (2) a decrease in the number of macrophages. A reduction in the number of pathogenic bacteria was also detected. The analyses of tumour necrosis factor-alpha, interleukin-10, and beta-defensin-2 revealed that the production of the aforementioned cytokines significantly increased, whereas no significant effects on interleukin-1 and caspase-6 expression in milk leukocytes were recorded. However, there were significant differences between mammary glands with high and low milk somatic cell count. The results suggest that the composition GLP 810 has an immunomodulatory effect on mammary glands and it could be used for improving the immune response in cows with subclinical mastitis during lactation.

## 1. Introduction

Mastitis or udder inflammation is a disease of major economic importance in dairy industry worldwide as discussed elsewhere [1, 2]. Subclinical mastitis is a particular condition of the mammary gland characterized by a long-term low-intensity infection and subsequent damage of the mammary gland tissue resulting in changes to milk composition. The animal immune status plays a key role in the development of mastitis after an invasion of bacteria into mammary glands; therefore, the maintenance of animal health by encouraging the natural immunological defence including the use of immunomodulators could serve as an important alternative to antibiotic therapy. Predominantly, the treatment of mastitis is still based on the use of antibiotics, but, according to Gyles [3] and Persoons with coauthors [4], the overall cost of this treatment is fairly expensive, whilst its effectiveness is constantly decreasing and is associated with significant harm to public health since the presence of residues in milk can promote development of antibiotic resistance.

The fight against the development of bacterial resistance is based on prudent and rational use of antibiotics, and it is a priority task in the human health sector and veterinary medicine worldwide, but alternative methods for the treatment of mastitis are needed to carry out this task. The authors have shown that the use of natural substances such as lactoferrin [5], nisin [6], lacticin [7], and probiotic lactobacilli [8–10] is also effective in the treatment of bovine mastitis. The aim of this study was to investigate the effect of intramammary infusions of natural composition GLP 810 with immunomodulating properties on the local nonspecific cellular and humoral immune response in cows with subclinical mastitis.

## 2. Materials and Methods

### 2.1. Composition GLP 810

The immunomodulatory composition GLP 810 (PCT patent WO2017010856A1) used in this study was prepared at the Institute of Microbiology and Virology of Riga Stradins University, and the following ingredients were included in each single dose of this preparation: lactic acid 500 mg, lysozyme 300 mg, glycopeptide from *Lactobacillus helveticus* 10 mg, and 0.9% sodium chloride solution in water 10 mL.

### 2.2. Animals and Experimental Design

Ten Holstein and Latvian Brown dairy cows in the middle lactation period of their 2^nd^ or 3^rd^ parity were used. Their average 305-day yield was 8,000 kg. The herd consisted of 320 dairy cows housed in tie-stall arrangement and milked twice a day. The cows involved in this study were subclinically infected in one or two mammary glands—six out of nineteen lactating mammary glands in each cow group had mastitis causing elevated somatic cell count (SCC) greater than or equal to 400,000 per mL in each individual cow's composite milk. All the cows included in this study had an increased somatic cell count at least for two consecutive months. No cow had received any drug-related intervention at least 30 days prior to the treatment start date. No cow had a history of concurrent disease other than subclinical mastitis and had no clinically significant findings. On the start of the study, no cow had any visible change in visual milk properties. The unit of study was mammary gland. Nineteen mammary glands in the experimental cow group were infused with 10 mL of composition GLP 810 three times at 48 h intervals (1^st^, 3^rd^, and 5^th^ days). Nineteen mammary glands in the control cow group were treated with 10 mL of 0.9% NaCl solution at the same time as the experimental group. Infusions were carried out in the morning after milking from 4 : 00 to 6 : 00 a.m. The clinical assessment of cow health status was carried out daily from the 1^st^ to the 7^th^ days, as well as on the day 14, marking the changes in the following parameters: systemic changes (body temperature, milk yield, and appetite), clinical appearance of the mammary glands (redness, hardness, pain, swelling, and heat), and the visual appearance of the milk (flakes, clots, and watery appearance). Both the control solution and the immunomodulatory composition were infused through the teat canal using an individual single-use teat cannula J-12 (Jorgensen Laboratories, USA) for each mammary gland. Before the infusion, the teat was scrubbed with a pad soaked in 70% ethanol; then, four to six streams of milk were drawn and discarded, and disinfection was repeated. After the infusion of the solution, udder massage in the upward direction was carried out to facilitate an even dispersal of the solution into the mammary gland. Teats were dipped in an iodine disinfectant solution by performing routine udder hygiene measures. Quarter milk samples from all cows were aseptically collected from each mammary gland 5 times, i.e., prior to the treatment (1^st^ d) and on the 3^rd^, 5^th^, 7^th^, and 14^th^ days after the first infusion. In total, 426 milk samples for this study were obtained and the following examinations were performed: bacteriological examination, differential counting of somatic cells, immunocytochemical analysis, and milk quality studies.

The study was restricted to routine on-farm observations and measurements, including milk sample collection that did not inconvenience or stress the animals. The composition GLP 810, that was used, included solely registered ingredients.

### 2.3. Bacteriological Examination

Microbiological examinations were carried out at the Microbiology Laboratory of the Institute of Food and Environmental Hygiene of the Latvia University of Life Sciences and Technologies, Faculty of Veterinary Medicine (LLU VMF). Bacterial examination analyses included evaluation of the total number of mastitis-causing (pathogenic) bacteria and bacterial clearance, i.e., absence of growth of the bacteria in milk.

Enumeration of staphylococci was performed in accordance with the standard LVS EN ISO 6888-1:1999/A1:2003 “Microbiology of food and animal feeding stuffs—Horizontal method for the enumeration of coagulase-positive staphylococci (*S. aureus* and other species)—Part 1: Technique using Baird-Parker agar medium—Amendment 1: Inclusion of precision data”. We confirmed the identification of *S. aureus* with the diagnostic reagent “Staphytect Plus” (Oxoid, UK), where a positive reaction (confirmed *S. aureus*), according to the manufacturer's instructions, was a formation of macroscopically visible flakes. For isolates of coagulase-negative staphylococci, identification was carried out to a species level using BBL Crystal Gram-Positive ID (Becton, Dickinson and Company, USA) and MALDI Biotyper (Bruker, DE) as per the manufacturer's instructions. The enumeration of bacteria of the family *Enterobacteriaceae* was carried out by a pour plate technique in accordance with the standard LVS ISO 21528-2:2007 “Microbiology of food and animal feeding stuffs—Horizontal methods for the detection and enumeration of *Enterobacteriaceae*—Part 2: Colony-count method”. *E. coli* detection was carried out in accordance with a previously described technique [11]. Isolation of *Streptococcus* and *Enterococcus* spp. bacteria was performed following UK Standards for Microbiology Investigations [12]. Additionally, the PathoDxtra Strep Grouping Kit for the Lancefield group (A, B, C, D, F, and G) *Streptococcus* spp. identification was used according to the manufacturer's instructions (Oxoid, UK). The determination of lactic acid bacteria (*Lactobacillus* spp.) was performed according to the method described by the authors [11]. This method is based on the requirements of standard ISO 15214: 1998 “Microbiology of food and animal feeding stuffs—Horizontal method for determination of mesophilic lactic acid bacteria—Colony count method at 30°C”.

### 2.4. Differential Counting of Somatic Cells

Differential counting of the milk smears was performed according to the methodology described by the authors [13] to specify the belonging to leukocyte populations. This investigation also was conducted at the Microbiology Laboratory of the Institute of Food and Environmental Hygiene of the LLU VMF. Each smear was examined by light microscopy at 400 and 1000 times magnification, counting 100-200 cells and determining their belonging to leukocyte populations: lymphocytes, macrophages, and polymorphonuclear leukocytes (PMNL).

### 2.5. Immunocytochemical Analysis

A customized biotin-streptavidin immunocytochemical method was performed as described by Hsu et al. [14] to evaluate the expression of inflammatory cytokines interleukin-1 (IL-1) and tumour necrosis factor-alpha (TNF-*α*), inflammatory-inhibiting cytokine interleukin-10 (IL-10), and antibacterial peptide beta-defensin-2 (BD-2) expression, as well as apoptosis marker caspase-6 in milk. A preparation of a specimen and placing of milk sediment on glass slides was performed at the Microbiology Laboratory of the Institute of Food and Environmental Hygiene of the LLU VMF, but a staining of the smears was carried out at the Morphology Laboratory of the Institute of Anatomy and Anthropology at Riga Stradins University. The following primary antibodies were used for immunocytochemical staining of the smears: rabbit polyclonal IL-1*α* (code AB17281, dilution ratio 1 : 200, Abcam, UK) and IL-10 (code Ab34843, 1 : 400, Abcam, UK), goat polyclonal beta-defensin-2 (code AF2758, 1 : 100, RD Systems, UK), rabbit monoclonal caspase-6 (code Ab52951, 1: 250, Abcam, UK) and rabbit polyclonal tumour necrosis factor-alpha (code ab6671, 1 : 100, Abcam, UK), and biotin-related secondary antibodies (LSAB+LINK, code K1015, *DakoCytomation*, DK).

### 2.6. Milk Quality Studies

The examination of milk quality was carried out at Dairy Laboratory Ltd. (Ulbroka, Latvia). The number of somatic cells was determined by fluorooptoelectronic cell counting using a Fossomatic FC somatic cell counter (Foss A/S, DK) in accordance with the standard LVS EN ISO 13366-2:2007 “Milk—Enumeration of somatic cells—Part 2: Guidance on the operation of fluoro-opto-electronic counters”. The determination of the total bacterial count was carried out with the electronic device “BactoScan FC” (FOSS Electric AS, DE). The determination of fat, lactose, and protein content in milk (%) was carried out using the infrared spectroscopy method in accordance with ISO Standard 9622 :1999 “Whole milk. Determination of milk fat, protein and lactose content-Guidance on the operation of mid-infrared instruments.” The pH was measured using a pH meter; the working principle of which is to measure the electrical conductivity of the milk and display the result converted into the corresponding pH value.

### 2.7. Statistical Analysis

Results were expressed as average ± standard error. Comparisons of the dynamics of mean parameters were performed by the Wilcoxon Signed Ranks two-sample test. The Mann–Whitney *U* test was used for comparisons between GLP 810 and control group parameters. We also used a Chi-Square test to independently assess whether two variables differ from each another. The Cramer's *V* test allowed us to assess the strength and relevance of the relationship between two features that did not fit the normal distribution or between two scale variables. In contrast, we used the Kendall's (Kendall's tau-b) correlation coefficient to measure the relationship between two ordinal variables [15, 16]. For assessment of the expression of cytokines, BD-2, and caspase-6, the semiquantitative counting method for a relative quantity of positive structures [17] was used. The positive structures according to their quantity into immunoreactive cells were divided into several groups: (-) none, (+) a few, (++) average, and (+++) a lot. To compare distribution of results obtained by means of immunocytochemical methods, a relative frequency calculation model was used [18]. Statistical significance was set at *p* < 0.05 for all statistical analyses.

## 3. Results

### 3.1. Leukocyte Dynamics in Milk

Using milk leukocyte differential counting, a significant increase in the number of polymorphonuclear leukocytes (PMNL) was observed that occurred both in the milk of the experimental (about 56-63%, *p* < 0.001) and the control groups (about 45-63%, *p* < 0.01, *p* < 0.05) following intramammary infusions, as shown in Table 1. The increase in PMNL count continued until the 14^th^ day and probably longer in the experimental group mammary glands, while the number of PMNL was decreased on the 14^th^ day in the control group milk with no significant difference from the baseline.

**Figure 1:**
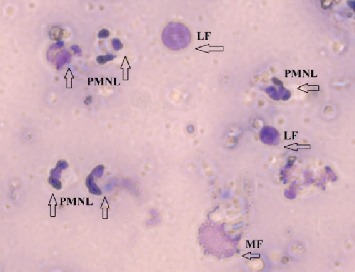
Microscopic examination of milk somatic cells smear. PMNL: polymorphonuclear leukocytes; LF: lymphocytes; MF: macrophages; original magnification 1000x.

As shown in Table 1, the number of lymphocytes increased significantly in the milk of the experimental group (*p* < 0.001), whereas no significant changes (*p* > 0.05) in the lymphocyte count were detected in the control group mammary glands. The number of macrophages after infusions diminished both in the milk of experimental (*p* < 0.001) and control groups (*p* < 0.01). It was supposed that the absolute number of macrophages had not changed noticeably, because their percentage fraction had decreased due to the parallel increase in PMNL and lymphocytes.  Figure 1 shows a typical view of milk smears of cows with high SCC on the 3^rd^ day of the study.

In summary, after GLP 810 infusions, the alterations in milk's PMNL count (increase by 50-60%) as well as lymphocyte (increase by 60%) and macrophage (decrease by 65%) count lasted during the entire observation, i.e., from the 3^rd^ day to the14^th^ day. In the control group, the significant changes in the distribution of cells (where the number of PMNL had increased by 50-60% and the number of macrophages decreased by 30-75%) lasted until the 7^th^ day but had returned to baseline by the 14^th^ day.

### 3.2. Expression of the Inflammatory Cytokines

Prior to the infusions, the TNF-*α* immunoreactive cells, mainly macrophages, were found in the milk of both cow groups. These cells were found to be present in small amounts at similar frequencies (16-25%) without any correlation to the number of SCC and pathogenic bacteria. As shown in Table 2, as soon as the infusions of GLP 810 were started, the expression of TNF-*α* in experimental mammary glands showed a general tendency to increase by 2.3 times and in the following period from the 3^rd^ to the 7^th^ day remained unchanged.

**Figure 2:**
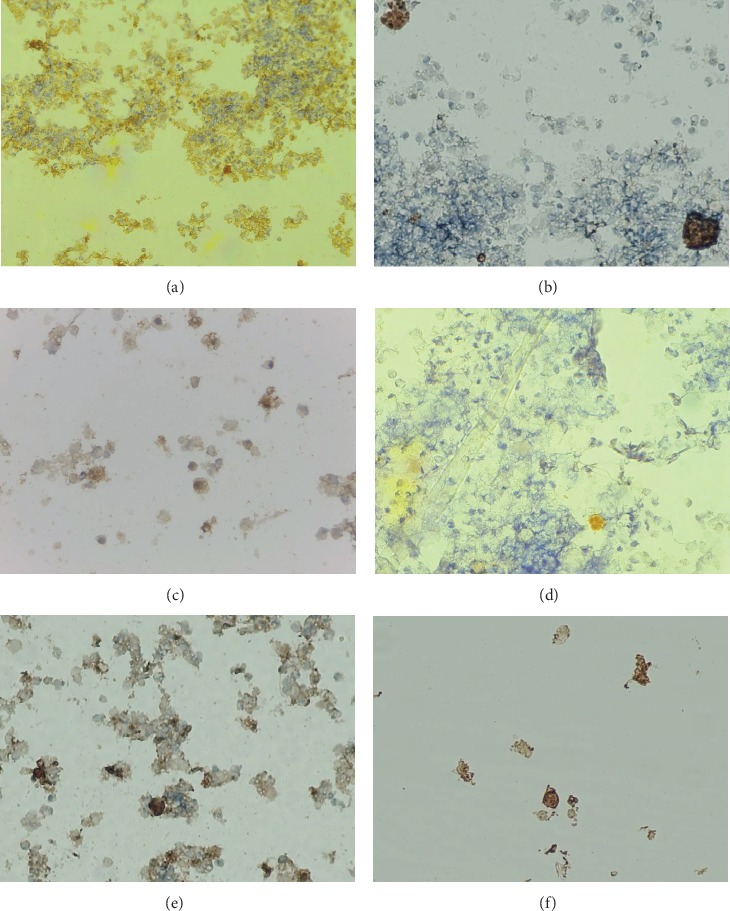
Expression of cytokines in milk by biotin-streptavidin immunocytochemistry method: (a) TNF-*α*, 100x; (b) TNF-*α*, 400x; (c) IL-1, 400x; (d) IL-10, 400x; (e) BD-2, 400x; (f) caspase-6, 400x. The brown colour in the cytoplasm and cell nuclei indicates a positive presence of cytokines.

The expression of TNF-*α* (see Figure 2) was significantly higher in the samples with high SCC and high number of pathogenic bacteria (more than 10 cfu × 10^3^ mL^−1^) than that in the samples with low SCC (*p* < 0.05). Besides, a TNF-*α* release from immune cells from the 1^st^ day was detected only in those milk samples, where somatic cells comprised more than 10% of PMNL and less than 10% lymphocytes.

After infusions of composition GLP 810, the expression of IL-1 in immune cells showed a tendency to decrease gradually, and on the 7^th^ day, the IL-1 was found to be approximately 80% less in the baseline group (see Table 2). Throughout the study, IL-1 was detected only in those milk samples of the experimental group, where *S. aureus*, *Staphylococcus* spp. and bacteria belonging to the family *Enterobacteriaceae* including *E. coli* were isolated (1 d Cramer's *V* coeff. 0.827 and *p* < 0.005; 3 d Cramer's *V* coeff. 0.659 and *p* < 0.05). In contrast, when the pathogens were not isolated, no expression of IL-1 in cells was detected.

As shown in Table 2, the expression of BD-2 in terms of frequency and intensity in leukocytes in the milk of the experimental group had already increased on the 3^rd^ day, but a significant increase was found on the 7^th^ day, when milk secretion of 80% (15/19) of the mammary glands had been disturbed and most of the milk samples (18/19) contained BD-2-positive cells (*p* < 0.01). BD-2-positive immune cells in variable frequency in the milk samples of the control group were also found, but the observed changes were statistically insignificant.

The frequency and intensity of IL-10 expression in the milk samples of the experimental group after GLP 810 infusions increased significantly (on the 3^rd^ day, *p* < 0.05; on the 7^th^ day, *p* < 0.005) until each of the 19 experimental group milk samples contained IL-10-positive cells on the 7^th^ day. The expression of IL-10 showed a decreasing trend (see Table 2) in the milk samples of the control group, and on the 7^th^ day, it was significantly lower in comparison with the milk samples of the experimental group (*p* < 0.05). The expression of IL-10 on the 1^st^ day was significantly higher in the milk samples with high SCC (Kendall test 0.774 and *p* < 0.001).

As shown in Table 2, the change in caspase-6 expression was low and statistically insignificant (*p* > 0.05) in both study groups. Both the experimental (*p* < 0.005) and control groups (*p* > 0.05) showed an expression of caspase-6 only in those milk samples where the SCC was more than 200 × 10^3^ cells mL^−1^. A statistically significant close relationship was also observed between the amount of caspase-6-positive cells and the number of PMNL in milk, because obviously more leukocytes meant more caspase-6 expression in the leukocytes (in the GLP 810 group *p* < 0.005, in the control group *p* < 0.05). Throughout the study, the release of this enzyme in both study groups was found only in those milk samples where the number of PMNL was at least 30% of countable cells.

### 3.3. Bacteriological Examination

Prior to administration of the composition GLP 810 in mammary glands, mastitis-causing bacteria were present in 84.2% (*n* = 16/19) of all experimental group mammary glands and in 47.4% (*n* = 9/19) of control group mammary glands. Detected pathogens belonged to *S. aureus* and other bacteria of the genus *Staphylococci* and the bacteria from *Enterobacteriaceae* family, including *E. coli*., GLP 810 infusions on the 3^rd^ study day provided full bacteriological clearance in 10 out of 16 infected mammary glands (62.5%). Bacterial clearance in the control group on the 3^rd^ day was detected in 4 out of 9 infected mammary glands (44.4%). On the 7^th^ study day, bacteriological clearance was diagnosed in 8 initially infected mammary glands of the experimental group (50.0%) and in 5 mammary glands of the control group (55.5%) which was a nonsignificant difference.

Furthermore, the total number of pathogenic bacteria was examined. As it is shown on Table 3, a significant decrease in the total number of pathogenic bacteria on the 3^rd^ day by 87% (*p* < 0.05) and on the 7^th^ day by 92% (*p* < 0.05) in the GLP 810 group mammary glands was observed.

Throughout the study, the total number of pathogenic bacteria did not change remarkably (*p* > 0.05) in initially low SCC and control cow group mammary glands, whereas the significant decrease in the number of pathogenic bacteria by 97% (*p* < 0.05) was detected in initially infected experimental cow group mammary glands with high SCC.

**Figure 3:**
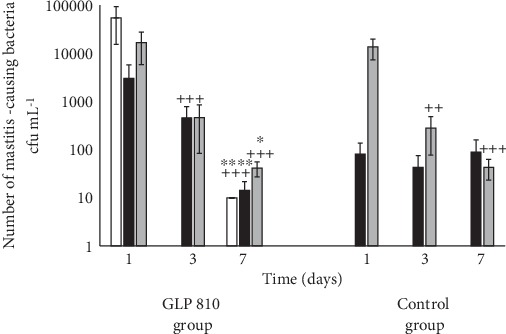
Dynamics of the number of mastitis-causing bacteria in milk: *S. aureus* (white bar), *Enterobacteriaceae* (black bar), *Staphylococcus* spp. (gray bar); ++*p* < 0.01, +++*p* < 0.001 versus baseline value; ^∗^*p* < 0.05, ^∗∗∗^*p* < 0.001 versus 3^rd^ day value; error bars indicate 95% confidence intervals of the mean values.

As shown in Figure 3, the number of *S. aureus* bacteria in the milk of the experimental group (*n* = 4) on the 1^st^ day averaged 54.3 ± 7.9 cfu × 10^3^ mL^−1^. Two days after the first infusion of the composition GLP 810, *S. aureus* was not detected in any milk samples, but on the 7^th^ day, a low amount of this pathogen was isolated only in one milk sample (10.0 cfu mL^−1^-99.9% less than on the 1^st^ study day).

The average number of *Enterobacteriaceae* microorganisms is significantly reduced only in the milk samples of the GLP 810 study group (*p* < 0.001), but the average number of *Staphylococcus* spp. is significantly decreased in the milk both in the experimental (*p* < 0.001) and in the control groups (*p* < 0.001, *p* < 0.01) (see Figure 3).

### 3.4. Evaluation of Milk Quality Indicators

**Figure 4:**
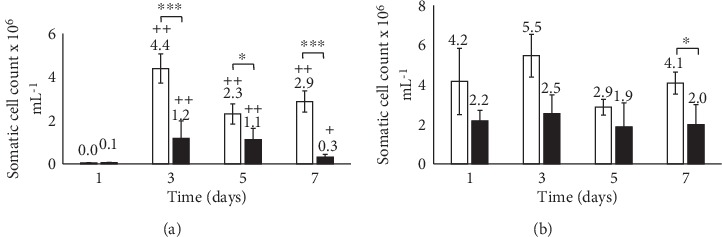
Dynamics of somatic cell count in the milk from healthy (a) and inflamed (a) mammary glands. GLP 810 group (white bar), control group (black bar); ^∗^*p* < 0.05, ^∗∗∗^*p* < 0.001 (between study groups); +*p* < 0.05, ++*p* < 0.01 (versus baseline value); error bars indicate 95% confidence intervals of the mean values.

In general, a rapid increase in the SCC was detected in the milk of the experimental group 48 hours after the 1^st^ and 3^rd^ infusions of GLP 810 while SCC dynamics in the control group mammary glands was statistically insignificant throughout the study period. The increase in SCC occurred mainly in healthy mammary glands (with low SCC on the 1^st^ day) (*p* < 0.01) both in the experimental and the control study groups, while the SCC did not change remarkably in the milk from inflamed mammary glands as shown in Figure 4.

**Figure 5:**
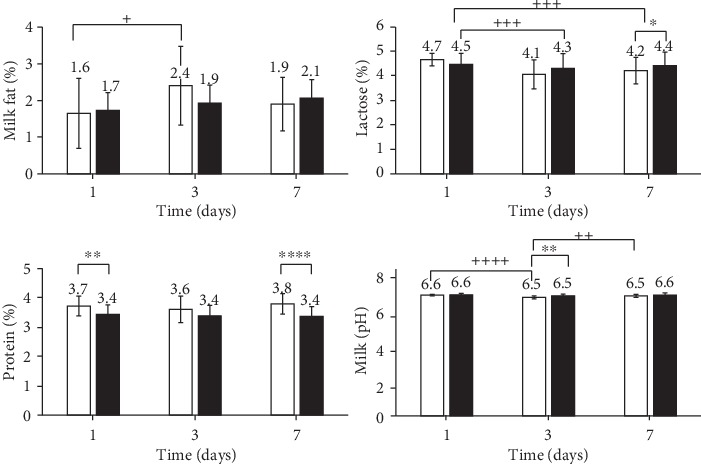
Milk fat, lactose, protein, and pH dynamics in milk. GLP 810 group (white bar), control group (black bar); ^∗^*p* < 0.05, ^∗∗^*p* < 0.01, ^∗∗∗^*p* < 0.001 (between study groups); +*p* < 0.05, ++*p* < 0.01, +++*p* < 0.001 (between days); error bars indicate 95% confidence intervals of the mean values.

The amount of lactose in the milk of the GLP 810 study group significantly decreased from 4.7% on the 1^st^ day to 4.1% on the 3^rd^ day, and on the 7^th^ study day, the lactose content was still significantly reduced (*p* < 0.001), i.e., by 4.2% (see Figure 5). The milk protein content after infusions did not change significantly. The milk fat content in the experimental group had significantly increased by the 3^rd^ day (from 1.6% to 2.4%; *p* < 0.05), but the control group expressed no significant change (see Figure 5).

### 3.5. The Bovine Health and Changes in the Milk Consistency

Visual changes in the milk consistency, i.e., a small number of flakes or clots, were observed every day during the first week of the study. Significantly more frequent changes were found to be in the milk obtained from the experimental group cows from the 3^rd^ to the 6^th^ day of the study. It was found that the formation of flakes and clots was significantly influenced by the milk pH value (*p* < 0.005) since they were only observed in milk samples with a pH range of 6.40-6.52, that is, lower than the average in the group. In addition, the changes in the milk consistency were probably due to the increased activity of leukocytes in the milk, as almost all of the milk samples (19/20), that contained the clots and flakes, had also high SCC (more than 200 × 10^3^ cells mL^−1^) (*p* < 0.001).

## 4. Discussion

The protection of the mammary gland against infections is provided by coordinated cooperation between nonspecific or innate and specific or acquired immune mechanisms. Consequently, the milk contains, either permanently or temporarily, a variety of cellular and humoral components that are involved in the immune response, thus making milk a biologically active product. In order to evaluate the effect of the immunomodulatory composition GLP 810 on the functional state of the bovine nonspecific cellular immune system locally in the mammary glands, we evaluated the number of leukocyte populations. The effect of the composition GLP 810 on the number of leukocytes and their populations as well as the lymphocyte subpopulation surface CD markers in the bovine peripheral blood is discussed previously by Gulbe with coauthors [19].

The present study revealed that applying intramammarily the immunomodulatory composition GLP 810 in cows with subclinical mastitis causes an activation of the local nonspecific cellular and the nonspecific humoral immune response, which results in limitation of growth of pathogenic bacteria in the mammary glands. In addition, we found that by GLP 810, the proposed immunomodulation was balanced, including the simultaneous migration of phagocytic cells to the udder tissue and the release of anti-inflammatory factors, providing controlled activation of the innate immunity. The observed immune activity included both the nonspecific cellular response and reactivity of nonspecific humoral factors between the 3^rd^ and 14^th^ days of the study.

Alnakip with coauthors stated [20] that leukocytes are permanently present in the mammary gland environment as they aid in the restructuring of the udder tissue during involution (i.e., apoptosis) or after inflammation and also provides immediate immune response in case of invasion of pathogenic bacteria while PMNL are the first recruited immune cells from blood to sites of infection in the mammary gland. As discussed elsewhere [21], the migration of PMNL during intramammary infection results in an increase of SCC with the aim to phagocytose the bacteria and to produce molecules of reactive oxygen and antimicrobial peptides capable to eliminate broad spectrum of mastitis causing bacteria.

As the significant increase in the number of PMNL occurred in the milk after the treatment with GLP 810, whereas the total number of leukocytes (as described on manuscript by [19]) and the number of segmented neutrophils (as shown in Table 3, [22]) in the peripheral blood did not significantly differ from the baseline and fell within the physiological norm throughout the study period in the experimental group, it may be concluded that the interaction of PMNL between the peripheral blood and the milk is indicative of the leukocyte-compensated proliferation in the mammary glands. On the other hand, the absolute number of segmented leukocytes in the blood of the control cow group had a significant increase on the 3^rd^ day exceeding the physiological norm while the relative amount of these leukocytes in the control milk exceeded the physiological norm at all times during the study [22].

Several authors [23–25] have shown that the phagocytic activity of the circulating blood neutrophils and macrophages increases after an application of probiotic lactic acid bacteria into mammalian organisms through their immunomodulatory properties. Probably, our study contradicts this fact, since the number of macrophages after infusions significantly diminished both in the milk of the experimental and the control groups. Furthermore, no remarkable changes in the total number of macrophage representative CD14^+^ cells were detected in the cow blood [19]. It is considered that the main function of macrophages is to develop substances that promote the local inflammation, thus stimulating phagocytic and bactericidal activity of PMNL, but phagocytosis is not the primary task of macrophages. The growing macrophage activity after infusions of GLP 810 is indicated by cytokine dynamics that is described further in the manuscript. Though we supposed that the absolute number of macrophages had not changed noticeably, their percentage fraction had decreased due to the parallel increase in PMNL and lymphocytes.

We observed that GLP 810 had a significant effect on lymphocyte activity *in vivo* indicated by the significant increase in the number of lymphocytes in the cow milk of the experimental group. Taking into account the results described previously [19], which the number of lymphocytes in the cow blood was significantly decreased after the start of GLP 810 infusions, it suggests a short-term noncompensated lymphocyte migration from the peripheral blood to the mammary gland.

Using milk leukocyte differential counting, we observed that the inflammatory reactions in the mammary glands were long-lasting because of the increase in PMNL and lymphocyte count, but, at the same time, the decrease in macrophage count lasted until the 14^th^ day after GLP 810 infusions.

To measure the progress of inflammation in the mammary glands, immunoreactive cells were labelled and the expression of nonspecific humoral factors—interleukin-1 (IL-1), tumour necrosis factor-alpha (TNF-*α*), interleukin-10 (IL-10), beta-defensin-2 (BD-2), and caspase-6—was identified in milk leukocytes.

As discussed elsewhere [21, 26–28], IL-1 and TNF-*α* are major inflammatory cytokines and are involved in both local and systemic immune responses through stimulating neutrophil leukocyte chemotaxis from the peripheral bloodstream to the mammary tissue where they arrive several minutes after the onset of infection. Bannerman [29] report that the presence of TNF-*α* was not detected in the healthy mammary glands.

At the start of the study, prior to the infusion of preparation, the TNF-*α*-immunoreactive cells, mainly macrophages, were found in the milk of both cow groups. These cells were found to be present in small amounts at similar frequencies (16-25%), without any correlation to a number of SCC and pathogenic bacteria. When starting the infusion of GLP 810, the expression of TNF-*α* in the experimental mammary glands increased in general by 2.3 times and in the following period from the 3^rd^ to the 7^th^ days remained unchanged at high levels, indicating the importance of this cytokine in maintaining inflammation; that is, maintaining PMNL activity, as the number of PMNLs in milk had significantly elevated during the period from the 3^rd^ to the 7^th^. It is known that neutrophils are the major producers of TNF-*α* during inflammation. We revealed that the expression of TNF-*α* was significantly higher in the samples with high SCC (average 3.4 ± 0.3 × 10^−6^ cells mL^−1^) than in the samples with low SCC (average 72.0 ± 8.4 × 10^−3^ cells mL^−1^). Besides, cytokine release from immune cells from the 1^st^ day was detected only in those milk samples where somatic cells comprised more than 10% of PMNL and lymphocytes—less than 10%. Such a leukocyte distribution in milk characterizes an infected mammary gland as demonstrated by Alhussien et al. [30].

IL-1 likewise TNF-*α* is an inflammatory cytokine, but IL-1 involvement in mastitis pathogenesis is not decisive and its significance is influenced by the type of infectious agent as discussed by Alluwaimi [27] and Shuster and Kehrli [31]. After infusions of GLP 810, the expression of IL-1 in immune cells was gradually reduced, and on the 7^th^ day, it was about 80% less than in the baseline readings. IL-1 release is thought to be reduced due to (1) a decrease in macrophages, (2) an increased anti-inflammatory cytokine IL-10 releases, and (3) an increased activity of an apoptosis.

The apoptosis in the mammary glands is indicated by the increase in the expression of caspase-6 immune-reactive cells on the 3^rd^ day by about 60% and by the decrease in the number of macrophages by an average of 65-70% from the 3^rd^ to the 7^th^ days. Other authors [27, 32] also confirm that the main distributors of IL-1 are monocytes in the blood and macrophages in the milk, but in a case of apoptosis, the amount of IL-1 decreases.

Beta-defensin-2 (BD-2) is characterized by bactericidal effects on pathogenic microorganisms and immunomodulatory action within both specific and nonspecific immunity. As previously described by Luenser and Ludwig [33] and Meade et al. [34], beta-defensins promote broad-spectrum leukocyte chemotaxis to the inflammatory site.

The observed increase in the expression of BD-2 in milk leukocytes after GLP 810 infusions suggests its significant role in the elimination of pathogenic bacteria, as well as in promotion of PMNL and lymphocyte migration to the mammary glands. The milk obtained on the 7^th^ day of research was characterized also by the lowest total number of conditionally pathogenic bacteria, the lowest number of *S. aureus*, and the bacteria from *Enterobacteriaceae* family and the *Staphylococcus* spp.

Asadullah et al. [35] and Bannerman [29] stated that IL-10 plays a key role in inhibiting inflammation and its action has a protective effect on limiting tissue damage and promoting tissue renewal after infections. We also detected the significant increase in the IL-10 expression in the milk of the experimental group. Furthermore, the role of IL-10 in reducing inflammation is confirmed by the fact that the expression of this immunity-regulating cytokine was significantly higher in the milk with elevated somatic cell count (Kendall coeff. 0.774, *p* < 0.001) and elevated total bacterial count (Kendall coeff. 0.676, *p* = 0.005). Our study proved that IL-10 activity in the cow mammary glands of the experimental group provided a balanced inflammatory response, which included the activation of the cellular immunity locally in the mammary glands, and lasted until the 14^th^ study day (or 9 days after the last infusion) resulting in a decrease in the number of pathogenic bacteria and number of somatic cell count.

During the study, we observed that after GLP 810 infusions, the number of pathogenic bacteria in milk samples significantly decreased by 87%–92% (*p* < 0.01) because of the antibacterial activity of the applied composition. The antibacterial activity of lactic acid, lysozyme, and probiotics was described elsewhere [8, 36–38], but studies are missing about the action of these natural substances *in vivo* after it is introduced in the udder. According to Panwar [39], Oliver and Wells [40], and Wang et al. [41], lysozyme and lactic acid cause physiological and morphological changes in bacterial cells that lead to bacterial growth inhibition and cell death. As the authors state, the hydrolysis products that originate from bacterial cell walls after lysozyme action are capable of enhancing immunoglobulin A secretion, macrophage activation, and rapid clearance of bacterial pathogens. And we hypothesized that also glycopeptides promote activation of immunity through macrophages, in order to activate T and B lymphocytes—the major cellular components of the adaptive immune response as it is described previously by Gulbe et al. (2016).

To evaluate the effect of immunomodulators on the quality of milk, analyses of the somatic cell count, pH value of the milk, and the amount of nutritional content (fat, lactose, and protein) were detected. Firstly, the rapid increase in the somatic cell count in the experimental group's milk after GLP 810 infusions was observed similarly as in the other studies in cows about intramammary application of bioactive preparations of *Lactobacillus* origin [42] and other substances with immunomodulatory properties such as TNF [43], recombinant bovine CD14 protein [44], and lactoferrin [5]. We found out that intramammary infusion with GLP 810 caused the increase in quarter milk SCC mainly because of an increase in SCC of initially uninfected quarters with low SCC (*p* < 0.01). The SCC dynamics in the control group mammary glands was statistically insignificant. Our observations are partially supported by the findings of Wellnitz et al. [45] that 0.9% NaCl infusion does not lead to a significant increase in quarter milk SCC.

Hence, a somatic cell count response after infusions of immunomodulatory substances is beneficial, even irreplaceable; the activity of leukocytes may result in decreased milk synthesis and the amount of milk-specific ingredients, as well as increased enzymatic activity of the milk, which is destructive to milk proteins and fats. However, our observations did not confirm either the loss of milk synthesis or the decrease in the amount of milk components after the infusion of preparations, except for the amount of lactose. Considering that infusions of GLP 810 did not lead to a destruction of nutrients in the milk, we conclude that infiltration of neutrophils in the mammary gland tissues was of moderate intensity.

During the study, we did not find any deterioration in the clinical condition of cows (including body temperature, milk yield, and appetite), and the clinical state of mammary glands were without pathological changes (such as swelling, heat, hardness, redness, or pain). However, after infusions of GLP 810, a small number of flakes or clots were observed. Clots in the milk are usually the result of a degradation of the cellular structure of casein. The authors [46] report that casein loses its stability when the milk pH is at pH 4.6 or lower. However, our results do not confirm this possibility, since the pH value in any of the analysed milk samples was not lower than 6.2 and the protein content of milk did not change substantially during the study. It is possible that the formation of clots and flakes following the administration of the composition GLP 810 into mammary glands has led to somatic cell-derived proteases that can cause hydrolysis of beta casein and subsequent proteolysis [47]. In this case, however, the activity of proteases in the milk glands during the study has been low because it was observed only focally.

## 5. Conclusions

The results of this study have proved that the intramammary use of GLP 810 in cows can activate both the local nonspecific cellular and the humoral immune body response, which results in limitation of pathogenic bacteria growth in the mammary glands. In addition, we found that the immunomodulation proposed by composition GLP 810 was balanced and involved a simultaneous migration of phagocytic cells to the udder tissue and a release of anti-inflammatory factors, thus providing controlled activation of cellular immunity in the range of physiological norm between the 3^rd^ and 7^th^ days.

## Figures and Tables

**Table 1:** Dynamics of milk differential somatic cell count.

Milk somatic cells	Result	GLP 810 group (*n* = 19)				Control group (*n* = 19)			
		1^st^ d	3^rd^ d	7^th^ d	14^th^ d	1^st^ d	3^rd^ d	7^th^ d	14^th^ d
PMNL^c^	Mean number of cells, %	18.9	43.1	50.8	53.2	23.2	42.5	63.7	37.8
	Standard error of the mean	4.1	4.0	3.6	3.2	4.7	4.9	3.9	11.4
	*p* value versus baseline^a^	—	^∗∗∗^	^∗∗∗^	^∗∗∗^	—	^∗∗^	^∗^	>0.05
	*p* value between study groups^b^	>0.05	>0.05	>0.05	^∗^	>0.05	>0.05	>0.05	^∗^

Lymphocytes	Mean number of cells, %	15.0	34.2	29.1	27.3	15.7	17.8	21.7	16.3
	Standard error of the mean	3.4	2.7	2.3	2.4	1.7	2.7	2.8	2.2
	*p* value versus baseline^a^	—	^∗∗∗^	^∗∗∗^	^∗∗∗^	>0.05	>0.05	>0.05	>0.05
	*p* value between study groups^b^	>0.05	>0.05	^∗∗∗^	^∗^	>0.05	>0.05	^∗∗∗^	^∗^

Macrophages	Mean number of cells, %	66.1	22.7	20.0	19.5	61.2	39.8	14.7	46.0
	Standard error of the mean	4.8	2.9	3.8	2.6	5.9	4.8	4.9	10.5
	*p* value versus baseline^a^	—	^∗∗∗^	^∗∗∗^	^∗∗∗^	>0.05	^∗∗^	>0.05	>0.05
	*p* value between study groups^b^	>0.05	>0.05	^∗∗^	^∗∗^	>0.05	>0.05	^∗∗^	^∗∗^

^a^By Wilcoxon Signed Ranks test; ^b^by Mann–Whitney *U* test; ^∗^*p* < 0.05, ^∗∗^*p* < 0.01; ^c^PMNL: polymorphonuclear leukocytes; ^∗∗∗^*p* < 0.001.

**Table 2:** Cytokine expression in milk leukocytes.

GLP 810 group (*n* = 19)	TNF-*α*			IL-1		IL-10^∗^				BD-2^∗∗^				Caspase-6	
	—	+	++	—	+	—	+	++	+++	—	+	++	+++	—	+
	% of analysed samples														
1^st^ d	84	16	0	79	21	63	11	26	0	37	47	16	0	75	25
3^rd^ d	63	37	0	84	16	16	53	26	5	21	53	26	0	74	26
7^th^ d	63	37	0	95	5	0	37	58	5	5	47	37	11	89	11

Control group (*n* = 4)															
1^st^ d	75	25	0	100	0	50	25	25	0	25	50	25	0	75	25
3^rd^ d	75	0	25	100	0	50	50	0	0	50	25	25	0	75	25
7^th^ d	50	50	0	100	0	25	75	0	0	25	75	0	0	100	0

The positive structures according to their quantity in immunoreactive cells: (-) none, (+) a few, (++) average, and (+++) a lot. ^∗^Statistically significant differences in the GLP 810 group versus baseline value on the 3^rd^ day *p* < 0.05 and on the 7^th^ day *p* < 0.005; ^∗∗^statistically significant differences in the GLP 810 group versus baseline value on the 7^th^ day *p* < 0.005.

**Table 3:** Dynamics of the total number of pathogenic bacteria.

Result	GLP 810 group (*n* = 19)			Control group (*n* = 19)		
	1^st^ d	3^rd^ d	7^th^ d	1^st^ d	3^rd^ d	7^th^ d
Mean number of pathogenic bacteria, cfu × 10^3^ mL^−1^	50.7	6.8	4.1	13.2	9.8	18.2
Standard error of the mean	29.0	4.4	1.9	5.9	7.8	1.1
*p* value by Wilcoxon Signed Ranks test (versus baseline)	—	0.015^∗^	0.049^∗^	—	0.184	0.244
*p* value by Mann–Whitney *U* test (between study groups)	0.333	0.911	0.833	0.333	0.911	0.833

## Data Availability

The data (PDF file) used to support the findings of this study are available from the corresponding author upon request. This file contain descriptive information of studied cows (breed, age, milk yield, lactation, treatment) and laboratory data obtained before (day 1) and after (day 3, 5, 7, 14) treatment including results of milk bacteriological and cytological examination, milk content and cytokine profile.
